# Malignant Mesothelioma of the Tunica Vaginalis Testis—A Malignancy Associated With Asbestos Exposure and Trauma: A Case Report and Literature Review

**DOI:** 10.1177/2324709619827335

**Published:** 2019-03-15

**Authors:** AAM A. Baqui, Nicholas A. Boire, Tajruba T. Baqui, Dhanan J. Etwaru

**Affiliations:** 1 The Brooklyn Hospital Center, Brooklyn, NY, USA; 2 St George’s University, True Blue, Grenada; 3 St John’s University, Queens, NY, USA

**Keywords:** mesothelioma, asbestos, testicular

## Abstract

In this article, we report an unusual case of a malignant mesothelioma of the testis, presenting as hydrocele. The patient has a known medical history of trauma and occupational exposure to asbestos. The clinical features of this injury are discussed together with its immunohistochemistry. Surgical intervention is discussed due to the nature of this pathology.

## Introduction

Malignant mesothelioma of the tunica vaginalis of the testis is an exceedingly rare neoplasm but often fatal type of testicular malignancy that is often diagnosed only after surgery is performed. Since the description of the first case in 1957, less than 100 cases have been reported,^1-3^ and no guidelines have been established because of its rarity. Indeed, the tunica vaginalis originates from the same embryologic source as the visceral pleura, peritoneum, and pericardium.^4,5^ Mesotheliomas most commonly arise from the pleural or peritoneal cavity and less than 5% of cases arise from tunica vaginalis of the testis.^6^

Most patients initially present with painless scrotal enlargement, hydrocele being the most common presentation. Although potential risk factors such as chronic hydrocele, trauma, recurrent epididymitis, and herniorrhaphy have been noted and associated with malignant mesothelioma of the testes,^7,8^ asbestos exposure remains the risk factor most highly associated with this malignancy.^5,9^ Jones et al^10^ have found a positive occupational history in 41% of 27 reviewed cases, and Plas et al^9^ found a positive history of exposure in 34.2% of patients with mesotheliomas of the tunica vaginalis. In this article, we report a case of testicular mesothelioma of the tunica vaginalis that presented as a hydrocele, with a patient history of trauma and occupational exposure of asbestos.

## Case

**Figure 1.**
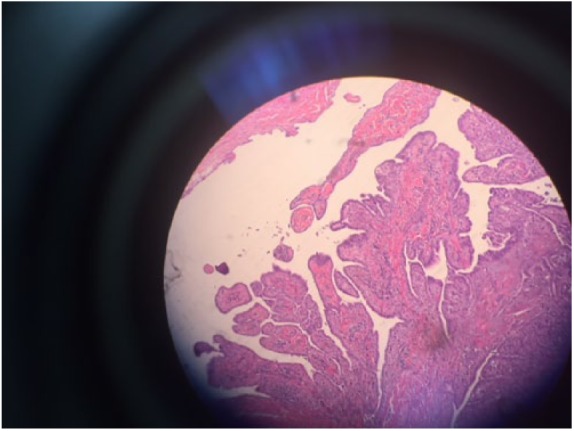
Low-magnification (40×) photomicrograph of a predominantly an exophytic papillary growth.

**Figure 2.**
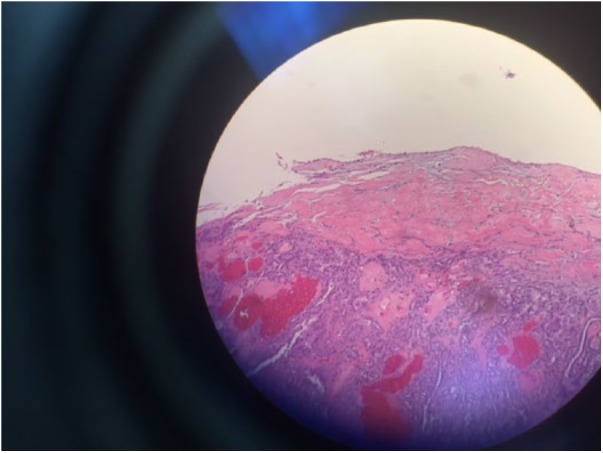
Low-magnification (40×) infiltration of the tumor into the tunica.

**Figure 3.**
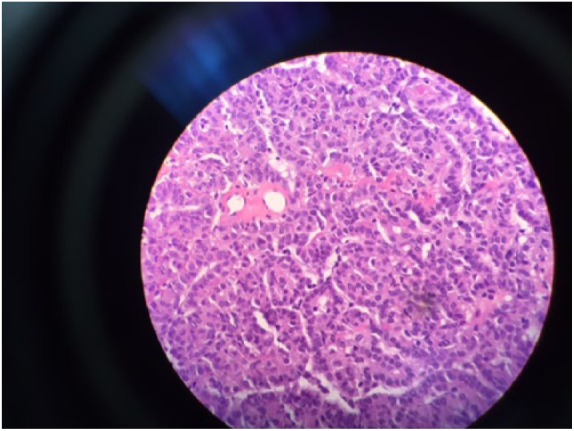
A relatively solid area of the tumor in high magnification (100×). Here the neoplastic cells are epithelioid with cuboidal to oval nuclei and eosinophilic cytoplasm.

We present the case of a 63-year-old male of African origin (Kenya) with an occupational work exposure to boilers, and with a known medical history of uncontrolled hypertension, diabetes mellitus type 2, brain aneurysm that developed after a motorcycle accident, and a large left testicular hydrocele (8 × 3 cm) developing for over 1 year. The patient was seen at another hospital with complaints of testicular enlargement but did not receive medical treatment at that time. More recently, the patient underwent a spectral and color Doppler ultrasound revealing a large complex left hydrocele with a 1.7 cm regular, heterogeneous solid structure within the upper anterior aspect possibly representing the left testis. Surgery was then performed a month later, 1300 cc of straw-colored fluid was noted, and the hydrocele sac and left testicular nubbin were removed via scrotal orchiectomy. After pathological review and consultation, the specimen was confirmed to be malignant mesothelioma, epithelioid type. The tumor demonstrates predominantly an exophytic papillary growth although in areas it is solid and invasive into the underlying tunica vaginalis. The neoplastic cells are epithelioid with cuboidal to oval nuclei and eosinophilic cytoplasm (Figures 1-3). By immunohistochemistry, the tumor cells are positive for WT-1, calretinin, and focally for D2-40, while BAP-1 is retained.

Follow-up for positron emission tomography/computed tomography scan did not reveal any hypermetabolic foci except for a small 9 mm hypodense nodule in the right lobe of thyroid gland.

Radical excision is planned and chemotherapy planned after more extensive surgery.

## Discussion

Radical inguinal orchiectomy with complete histologic examination and follow-up for an adequate duration is accepted as the optimal primary treatment option for malignant mesothelioma of the tunica vaginalis. Recurrence rate after radical orchiectomy ranges from 10.5% to 11.5% versus conservative surgical management such as local excision of the hydrocele having a 36% recurrence rate.^9^ The use of adjuvant therapy has not been studied well and remains inconclusive. Studies have repeatedly found that aggressive surgical management is the most important therapeutic approach in reaching complete remission and that chemotherapy or radiotherapy have not shown a clear benefit altogether. da Fonseca et al reported a case of malignant mesothelioma of testis treated postsurgically with pemetrexed and cisplatin with very dismal effect.^11^ Patient passed 24 days after chemotherapy.

In patients presenting with scrotal masses, and whether a history of exposure to asbestos is present or not, malignant mesothelioma should be a part of the differential diagnosis. Ultrasonography can give substantial information, and in case of suspicious cases cytoanalysis of the hydrocele fluid may be diagnostic. Once the diagnosis is established, usually in the postsurgical setting by pathology, aggressive surgical management should be the first line of treatment.
